# Neurosyphilis with a rare magnetic resonance imaging pattern confirmed by metagenomic next-generation sequencing: a case report

**DOI:** 10.1186/s40001-022-00676-1

**Published:** 2022-03-27

**Authors:** Chang Liu, Yu Zhang, Yi Li, Hui Bu, Ruoxue Liu, Yingxiao Ji, Junying He, Kun Hong

**Affiliations:** 1 Department of Neurology, The Second Hospital of Hebei Medical University, Shijiazhuang, China; 2 Institute of Cardiocerebrovascular Disease, Shijiazhuang, China; 3 Neurological Laboratory of Hebei Province, Shijiazhuang, China; 4 Department of Neurology, Hebei General Hospital, Shijiazhuang, China

**Keywords:** Neurosyphilis, Brain magnetic resonance imaging, Mesiotemporal abnormality, Metagenomic next-generation sequencing, Case report

## Abstract

**Background:**

Neurosyphilis refers to infection of the central nervous system by *Treponema pallidum*. The clinical presentation is variable and nonspecific. Neuroimaging findings are complex and that the diagnosis is based on clinical presentation, cerebrospinal fluid (CSF) parameters, and serologic and CSF evidence of syphilis. To date, there is no case report describing *Treponema pallidum* detected by metagenomic next-generation sequencing (mNGS) in CSF.

**Case presentation:**

In this report, we describe a case of neurosyphilis in a HIV-negative, 29-year-old man, who was admitted to our hospital with an epileptic seizure and progressive cognitive impairment. Brain magnetic resonance imaging (MRI) revealed fluid-attenuated inversion recovery (FLAIR) high signal intensities in bilateral medial and anterior temporal lobes, insula, right pulvinar of the thalami, precuneus, frontal and temporo-occipital lobes. Laboratory examination showed positive results by means of nontreponemal or specific treponemal test in serum and CSF. mNGS of the CSF was also performed to identify *Treponema pallidum* for the first time.

**Conclusions:**

This case underscores the importance of considering neurosyphilis as a potential cause of mesiotemporal abnormality. In addition, the rapid improvement and wide usability of mNGS technology will bring new breakthroughs in the clinical diagnosis of neurosyphilis.

## Background

Syphilis is known as the “Great Imitator” caused by the organism *Treponema pallidum*. The incidence of syphilis has increased in the past 20 years in China [1]. It is a sexually transmitted disease, and the clinical course can be divided into four stages; primary, secondary, latent, and tertiary syphilis. Neurosyphilis may occur during any stage of infection. The clinical presentation of neurosyphilis depends primarily on the location of involvement, and neurologic deficits may be subtle and are often overlooked [2]. Neuroimaging manifestations are challenging, include normal results of magnetic resonance imaging (MRI) findings, mild-to-moderate atrophy, white matter demyelination, cortical and subcortical infarction, cerebral gummas and leptomeningeal enhancement [3, 4]. Recently, it is clinically important to note that mesiotemporal hyperintensity on fluid-attenuated inversion recovery (FLAIR) MRI has been considered a probable rare finding in neurosyphilis [5–8]. Traditionally, the diagnosis of neurosyphilis is based on positive serum and cerebrospinal fluid (CSF) nontreponemal or treponemal tests, but until now no single laboratory test is both sensitive and specific. To our knowledge, *Treponema pallidum* was detected by metagenomic next-generation sequencing (mNGS) in the current report for the first time.

## Case presentation

**Fig. 1:**
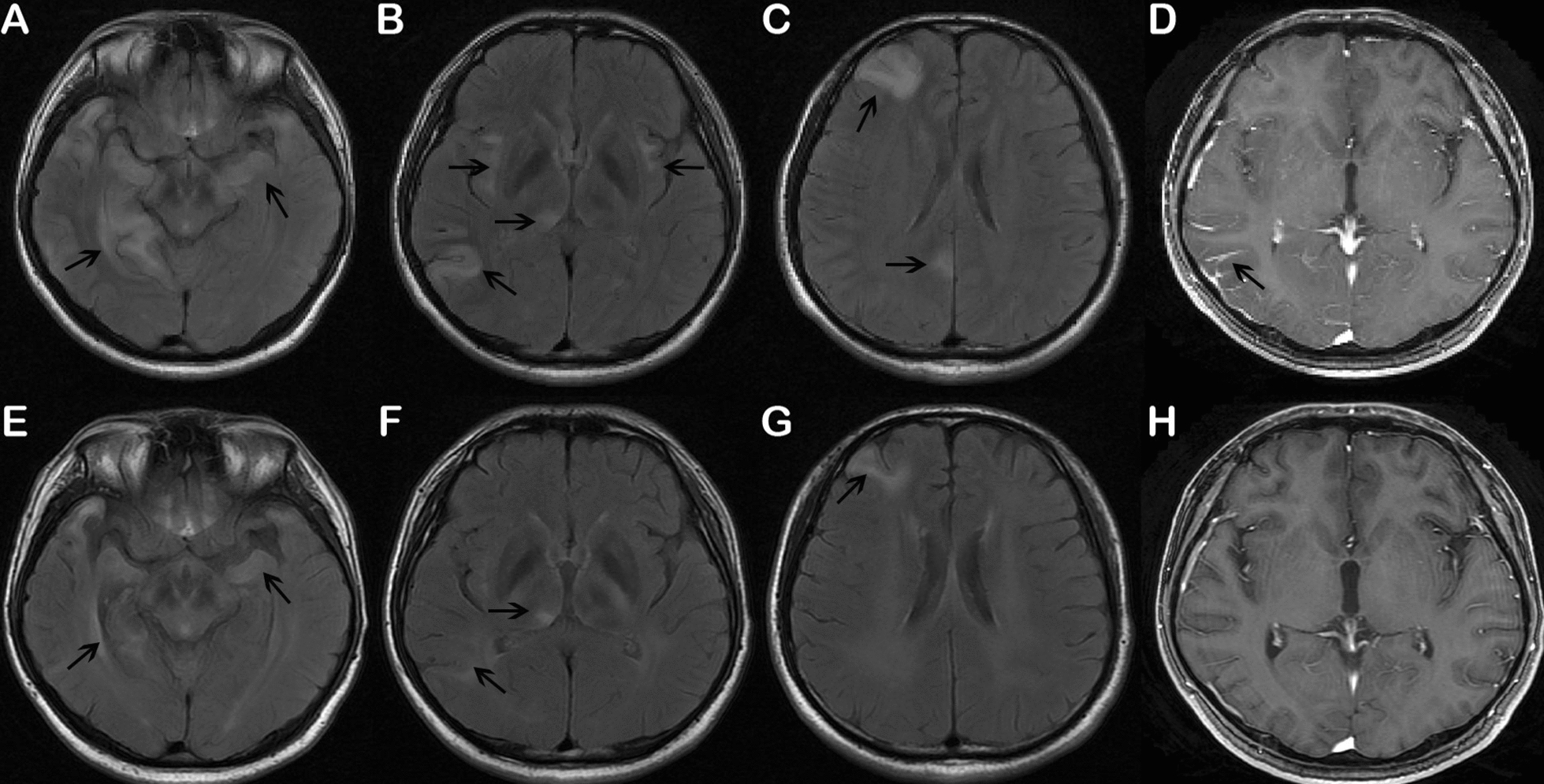
Initial and follow-up brain MRI findings. **A**–**C** Axial FLAIR images showed hyperintensities in bilateral medial and anterior temporal lobes, insula, right pulvinar of the thalami, precuneus, frontal and temporo-occipital lobes. **D** T1-contrast-enhanced MRI showed leptomeningeal enhancement in the right temporo-occipital region. **E**–**G** Post-treatment FLAIR images on 16th day of admission revealed marked improvement in the previously identified bilateral temporal, insula, precuneus, frontal and temporo-occipital hyperintensities. **H** Repeat gadolinium-enhanced MRI demonstrated disappearance of leptomeningeal contrast enhancement. The arrows indicated the lesions

**Fig. 2:**
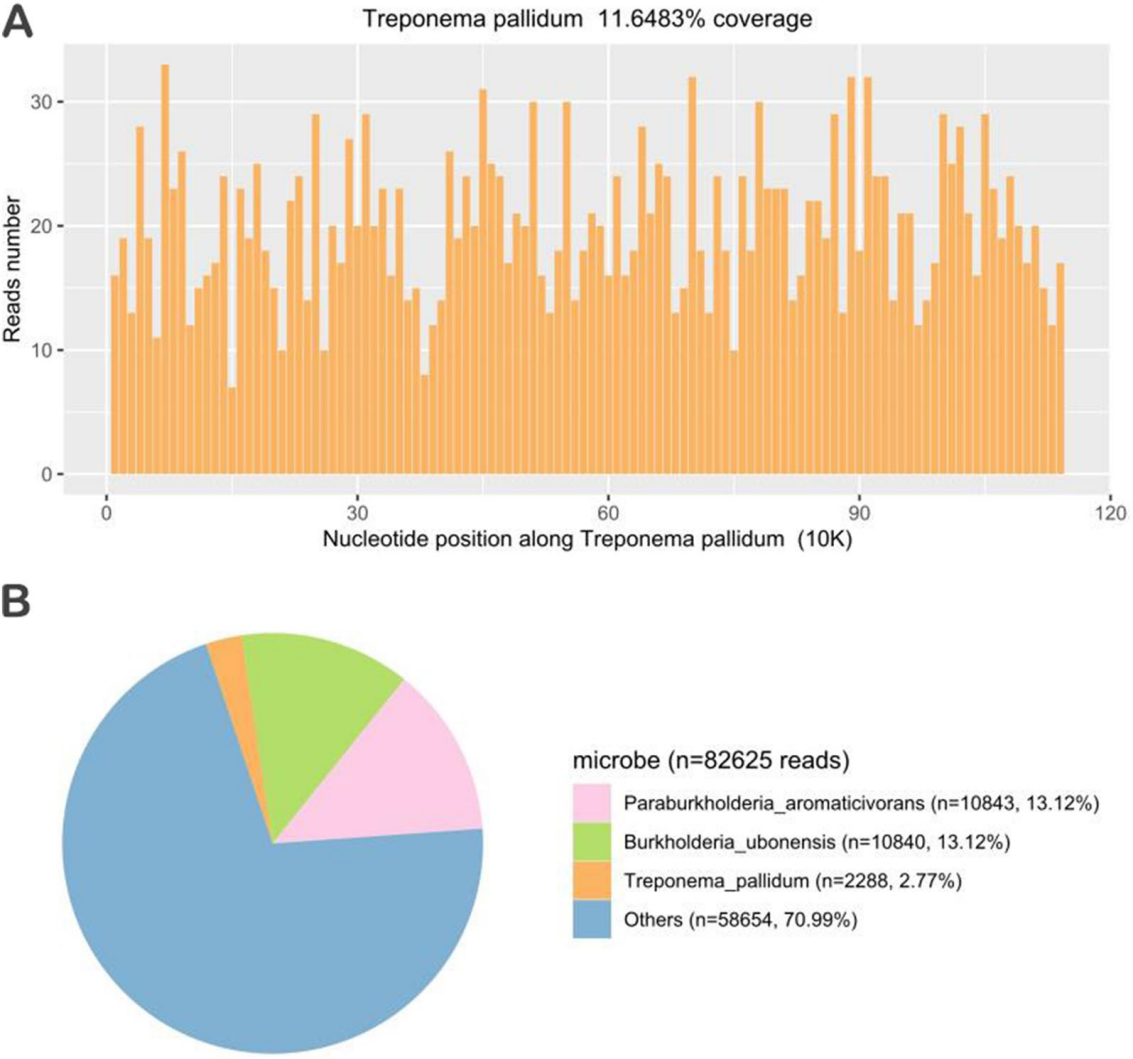
mNGS of *Treponema pallidum* in the patient’s cerebrospinal fluid. **A**, **B** The result of mNGS showed 2288 reads corresponding to the *Treponema* *pallidum*, with a coverage of 11.6483%. In the microbiota composition, 2.77% of the reads corresponded to *Treponema* *pallidum*, and the rest are commonly regarded as contaminating bacterial DNA from the environment and agents

A 29-year-old man was hospitalized with progressive memory impairment for 10 days. He was reported to struggle remembering different locations, especially home-base. His medical history included an epileptic seizure 3 months prior to admission, without early diagnosis and treatment. The parents denied that he had promiscuous sexual behavior with any other persons. On examination, an ulcer was noted on the anterior perineum. The patient was disoriented in space, and he did not even know the result of 93 minus 7. His Mini-Mental State Examination (MMSE) score was 22 out of 30. The remainder of his neurologic examination was normal. An electroencephalogram showed diffuse generalized slow spike-wave discharges. The blood work-up indicated a positive tolulized red unheated serum test (TRUST) with a titer of 1:32 and a positive fluorescent treponemal antibody-absorbed (FTA-ABS) test. Serum biochemistry, HIV and hepatitis serology, thyroid stimulating hormone, and glycated hemoglobin levels were unremarkable. Brain MRI showed high signal intensities on FLAIR images involving of bilateral medial and anterior temporal structures, insula, right pulvinar of the thalami, precuneus, frontal and temporo-occipital lobes, with focal meningeal enhancement (Fig. 1A–D), while MR-TOF angiography did not reveal any evidence of intracranial arterial stenosis. Lumbar puncture with CSF analysis was performed with an opening pressure of 15.5 cmH_2_O, which revealed pleocytosis (58 cells/µL), low glucose (2.18 mmol/L), and raised protein (1.39 g/L), his CSF TRUST also showed positive titres of 1:2. Furthermore, CSF sample was collected for mNGS to rule out co-infection. Interestingly, after 48 h, the results revealed 2288 sequence reads uniquely corresponding to the *Treponema* *pallidum* genome with 11.6483% coverage (Fig. 2A, B), confirming the diagnosis of neurosyphilis. Meanwhile, no other causative pathogen was identified. Following treatment with intravenous penicillin, 24 million units/day for two weeks, his memory and computation abilities improved greatly, and the MMSE score increased to 27 points. Repeat MRI of the brain performed on 16th day of admission showed shrinkage of FLAIR signal abnormalities within the right precuneus, frontal, temporo-occipital, and bilateral mesial temporal regions, without leptomeningeal enhancement (Fig. 1E–H). On the recent follow-up occasion, he was nearly a normal person and refused repeat testing, including lumbar puncture.

## Discussion and conclusions

Neurosyphilis is a chronic complication that is commonly found in adult with long latency periods, and up to 4–10% of patients with untreated syphilis may develop neurosyphilis [9]. According to clinical features and laboratory test results, it has classically been divided into early neurosyphilis (asymptomatic neurosyphilis, meningitis, meningovascular syphilis) and late neurosyphilis (tabes dorsalis, general paresis), however, there are some overlaps in clinical features between them [2]. In this report, the patient was diagnosed with general paresis based on the memory deficits, the disorientation and the positive findings in serum and CSF. General paresis is the commonest clinical presentation of neurosyphilis, in almost half of the patients, manifesting with cognitive impairment and psychiatric syndromes, often with seizures. Other features include personality change, manic delusions, tremor and dysarthria [2, 10]. Due to the lack of medical history and risky factors, we cannot conclude when our patient was initially exposed to syphilis. Symptomatic neurosyphilis usually occurs 1 to 2 years after infection, but can occur later [2]. Although general paresis may occur at any time in patients with syphilis, it tends to develop decades after the primary infection [10, 11].

MRI examination is imperative procedure for the detection of neurosyphilis, although two-thirds of patients are reported to have a normal finding, or a nonspecific mild-to-moderate cerebral atrophy. The other common diagnostic phenomenon includes cortical and subcortical infarction, white matter lesions, gummas, leptomeningeal enhancement, and cerebral vasculitis [3, 4, 12]. Non-contrast MR sequences (e.g., FLAIR; MR angiography) may also showed comparable diagnostic performance to contrast-enhanced MRI, as well as they are faster, more forgiving and less costly. Bilateral mesiotemporal involvement is not specific, which is considered as a typical sign of the herpes simplex encephalitis or autoimmune encephalitis. This abnormality is extremely rare in neurosyphilis, and we retrospectively analyzed the patients of neurosyphilis with this characteristic finding as reported in the literature from 2001 to 2020 [5–8, 13]. Of the 26 patients, 24 were men and 2 were women. Their ages ranged from 30 to 73 years, with a mean age of 48. Only 1 patient tested HIV-seropositive and 25 were HIV-seronegative. In addition to mesiotemporal abnormality, 5 patients with lesions involving frontal lobe and 1 patient with lesion involving thalamus were found. Parenchymal and focal meningeal enhancements were present in 2 and 3 patients, respectively. Surprisingly, no lesion was found in occipital or parietal lobe. Compared with previously published cases, our patient had more extensive lesions. The etiology and pathogenesis of mesiotemporal abnormality remain unclear. Some researchers have hypothesized that meningovascular inflammation causes cytotoxic and interstitial edema, besides, organism occludes small blood vessels, further results in anaerobic condition and gliosis in temporal lobe and limbic system [13, 14]. Meningovascular inflammation can be seen in both early and late stages of the disease [2], which may be observed at any distribution in the brain. As for our case, we speculate that it may be due to the activity of *Treponema pallidum* and individual factors, causing diffuse brain lesions.

Up to now, the diagnosis of neurosyphilis is usually based on combination of laboratory tests and clinical signs and symptoms. Laboratory diagnosis involved abnormal results of serum and CSF tests and on elevations in the CSF white cell count (> 5 cells/µl) and protein level (> 40 mg/dL) [11]. Serological tests consist of nontreponemal (Venereal Disease Research Laboratory [VDRL] or rapid plasma reagin [RPR] techniques) and treponema (FTA-ABS and related techniques) tests, which are imperfect and have inherent limitations. A reactive CSF nontreponemal test is highly predictive of neurosyphilis, although it is less than 80% sensitive. In contrast, the sensitivity of a positive CSF treponemal test is relatively high, but it lacks specificity [2, 11]. Additionally, Hagihara M et al. [15] emphasized that polymerase chain reaction (PCR) test should be preferred for the diagnosis of neurosyphilis, despite its lack of large-scale studies. In clinical practice, when patients develop increased intracranial pressure or syphilitic gumma, lumbar puncture should be performed cautiously.

There is increasing evidence that mNGS can play a role in neuroinfectious diseases, such as unexplained encephalitis and meningitis [16, 17], although it is difficult to used as a routine screening tool for its high cost. For cases of neurosyphilis with atypical symptoms or negative results by means of conventional testing, we believe that mNGS can be used as a supplemental diagnostic method. mNGS analysis of CSF allows for an unbiased and rapid approach to the detection of pathogens, which is also helpful for the differential diagnosis [16–18]. This is of importance, because the recent report, by Emmanuel et al. [19], was of a 51-year-old man co-infected with neurosyphilis and herpes simplex encephalitis. It is worth mentioning that a negative result reported by mNGS does not rule out neurosyphilis owing to the higher risk of false negative results [17]. Unfortunately, his family refused autoimmune encephalitis antibodies test. Given clear reduction of abnormal signal and clinical improvement following the 14-day course of penicillin, the final diagnosis of neurosyphilis was reached undoubtedly. In addition, It is necessary to perform follow-up neuroimaging in patients with neurosyphilis, because some patients still presented neuroradiological progression after standardized anti-syphilitic treatment [20].

In conclusion, misdiagnosis rate of neurosyphilis is still high due to atypical neuroimaging and clinical manifestations. Therefore, recognizing MR manifestations plays an important role in the diagnosis of neurosyphilis. We further emphasize the importance of early diagnosis and treatment in patients with mesiotemporal abnormality, because cognitive impairment resulting from neurosyphilis is considered to be “reversible dementia”. Despite its lack of sensitivity for use alone as a diagnostic test, mNGS should be preferred to offer a supplementary method for the diagnosis and differential diagnosis of neurosyphilis, and a larger number of cases will be needed to confirm this method.

## Data Availability

The data that support the findings of this study are available from the corresponding author upon reasonable request.
